# A comprehensive database of Duchenne and Becker muscular dystrophy patients (0–18 years old) in East China

**DOI:** 10.1186/s13023-014-0220-7

**Published:** 2015-01-23

**Authors:** Xihua Li, Lei Zhao, Shuizhen Zhou, Chaoping Hu, Yiyun Shi, Wei Shi, Hui Li, Fang Liu, Bingbing Wu, Yi Wang

**Affiliations:** Department of Neurology, Children’s Hospital of Fudan University, No.399, Wanyuan Road, Minhang District, Shanghai, 201102 China; Rehabilitation Department, Children’s Hospital of Fudan University, Shanghai, China; Cardiac Center, Children’s Hospital of Fudan University, Shanghai, China; Translational Research Center for Development and Disease, Children’s Hospital of Fudan University, Shanghai, China

**Keywords:** Duchenne and Becker muscular dystrophy, The CHFU database, Patient management

## Abstract

**Background:**

Currently, there is no cure for Duchenne and Becker muscular dystrophies (DMD/BMD). However, clinical trials with new therapeutic strategies are being conducted or considered. A comprehensive database is critical for patient recruitment and efficacy evaluation. China has the largest population, yet, no comprehensive database for DMD/BMD is available. Our study registered the data of the DMD/BMD patients in East China.

**Methods:**

A modified registry form of Remudy (http://www.remudy.jp/) was applied to Chinese DMD/BMD patients through the outpatient clinic at Children’s Hospital of Fudan University, Shanghai during the period of August 2011 to December 2013. The data included geographic distribution of patients, age at diagnosis, clinical manifestation, genetic analysis and treatment status.

**Results:**

194 DMD and 35 BMD patients were registered. Most patients lived in East China, namely Jiangsu province, Anhui province, Zhejiang province, Jiangxi province, Shanghai, Fujian province and Shandong province. All individuals aged less than 18 years (age limit to a children’s hospital). Diagnosis was made for a majority of patients during the age of 3–4 (16.6%) and 7–8 (14.8%) years old. Exon deletion was the most frequent genetic mutations (65.5% and 74.3%) followed by point mutations (14.4% and 11.4%), duplications (9.8% and 8.6%) and small insertion/deletion (9.3% and 2.9%) for DMD and BMD, respectively. 82.5% of DMD registrants were ambulatory, and all the BMD registrants were able to walk. 26.3% of DMD registrants have been treated with steroids. Cardiac functions were examined for 46.4% DMD boys and 45.7% BMD boys and respiratory functions were examined for 18.6% DMD boys and 14.3% BMD boys. Four boys with abnormal cardiac function were prescribed for treatment with cardiac medicine. 33.2% of DMD patients are eligible for exon skipping therapy, and among them 9.2% and 4.3% patients are eligible for skipping exon 51 and 53, respectively.

**Conclusions:**

The database is the first linking accurate genetic diagnosis with clinical manifestation and treatment status of dystrophinopathy patients in East China. It provides comprehensive information essential for further patient management, especially for promotion of international cooperation in developing experimental therapies such as exon skipping and read-through of nonsense mutations targeting a subgroup of DMD patient population.

## Background

Duchenne muscular dystrophy (DMD) and its milder form, Becker muscular dystrophy (BMD) are X-linked recessive muscular dystrophy caused by mutations in the dystrophin gene (DMD) on chromosome Xp21.2 [1,2]. The DMD gene is the largest gene identified in human and contains 79 exons. Patients with DMD have mutations that result in no dystrophin protein production, whereas those with BMD produce truncated but in-frame dystrophin with decreased quantity and function [3-5]. DMD is the most frequently inherited muscular disease, affecting 1 in 3600–6000 live male births [6]. It is characterized by progressive muscle weakness with onset at 3–5 years of age, leading to loss of ambulation by 10–12 years of age without treatment [7]. Respiratory, orthopaedic, and cardiac complications emerge with age, and without intervention, the mean age at death is around 19 years [6]. BMD has a slower rate of progression with time of loss of ambulation from adolescence onward to adulthood [8]. Some patients may continue walking into their fifties or sixties and have a normal life span [2,7].

Currently, although there is no effective treatment for DMD, the natural history of the disease can be improved by targeted interventions. Pharmacological and care-based interventions (corticosteroid, rehabilitative, cardiac, orthopaedic, respiratory, and psychosocial interventions) are proven to improve the muscle function, quality of life, and longevity. Children with DMD who are diagnosed today and receiving the generally recommended interventions have the possibility of a life expectancy into their fourth decade [6]. Moreover, promising therapeutic strategies are being developed in animal models and human trials of these strategies have started, leading to the hope of further dramatic improvement for the disease. These therapeutic strategies for DMD/BMD fall into four categories [9-12]: (1) mutation-specific therapeutic approaches for repairing the genetic defects or the transcripts (exon skipping, nonsense codon suppression and endonuclease-mediated gene correction); (2) gene replacement therapy, which can be applied to the entire patient population; (3) cell therapy including allogeneic stem cells with mutations corrected ex vivo; and (4) modulation of non-dystrophin gene expression such as upregulation of utrophin to compensate for the lack of dystrophin.

However, many of the therapeutic interventions target only a subset of the DMD population with defined genetic alteration. The best example is exon skipping. Each antisense drug can only target a small population of DMD with frame shift mutations correctable by the removal of specific exon(s). Jerry R. Mendell et al. reported that in prior open-label studies, eteplirsen, a phosphorodiamidate morpholino oligomer, enabled dystrophin production in DMD with genetic mutations amenable to skipping exon 51. 12 DMD boys participated in this study were reported with a 67.3 m benefit compared to placebo/delayed patients (p ≤ 0.001) [13]. Recently, Bushby et al. reported that Ataluren (Translarna™) offers promise as a treatment for DMD with nonsense mutation [14].

With limited number of DMD/BMD subpopulation, it becomes clear that, to meet general requirement by regulatory authorities, clinical trial of targeted therapy requires international collaboration to establish sufficient patient enrollment for therapeutic significance to be demonstrated statistically. Many countries have already established national databases and collaborated with the international registry of Treat-NMD [15]. However, awareness of the disease and development of experimental therapies in China lag far behind the industrialized nations both in the medical circles and society in general. Few medical institutions have specialized laboratories and physicians for diagnosis and treatment of DMD/BMD. In addition, research funds specific for the diseases from both private sectors and governmental organizations have been almost non-existent in China until very recently. This has led to a status where the epidemiological data and natural history of the disease of a large patient population are largely unmanaged. There is only one existing registry website set up in September 2012 by General Hospital of Chinese Armed Police Force, Beijing and China DMD care and support association (www.dmd-registry.com) and a DMD medical cooperation network has yet to be established in China. Clearly, a comprehensive database for Chinese DMD/BMD patients will be critical for care of these patients and for international efforts to develop experimental therapies. From August 2011, we started to establish a database for DMD/BMD patients in Children’s Hospital of Fudan University (named the CHFU database for dystrophinopathy). The database has registered all the DMD/BMD patients visiting the outpatient clinic of CHFU. Here we described the registry with genetic diagnosis, clinical manifestation and the status of treatment. Further expansion of the database in the country with the largest population in the world will be undoubtedly highly valuable for international cooperation to promote therapeutic research of the disease.

## Methods

### Institution and organization of the project

The CHFU database for dystrophinopathy is supported by Children’s Hospital of Fudan University. The development and management of the registry is led by the Neuromuscular research unit, department of neurology.

### Patients

The CHFU database includes DMD/BMD patients (all male Chinese) whose diagnosis has been confirmed by genetic analysis (see below section Phenotypic classification). Most registered patients were from provinces of East China, namely Jiangsu, Anhui, Zhejiang, Shanghai, Jiangxi, Fujian and Shandong.

### Method of registration and data collection

Information about the registry was provided to the patients diagnosed by genetic testing or muscle biopsy through DMD Outpatient Clinic in our hospital. Patient records were entered into the database on the basis of informed consent, which explained the content and aims of the database with voluntary registration. No identifiable personal data will be shared with any third party without the patient’s permission and the subject will be removed from the database immediately on request. This study was approved by Pediatric Research Ethics Board of Clinical Pharmacology Base, Fudan University.

### Structure of the registry form and patients’ follow-up

The registry form was designed with reference to the registry form of Remudy (http://www.remudy.jp/) [16]. Data from individual patient include name, gender, date of birth, address, phone number, family history related to muscular dystrophy, results of biochemical analysis (mainly serum creatine kinase levels) and muscle biopsy. Detailed description of genetic mutation was also included. Clinical symptoms include walking capability as well as cardiac and respiratory functions. Treatment including steroid therapy and history of scoliosis surgery was recorded. All data were validated by molecular and clinical curators. The database was semi-annually updated by outpatient visits for ambulant patients and through telephone follow-up for non-ambulant patients.

### Phenotypic classification

**Figure 1:**
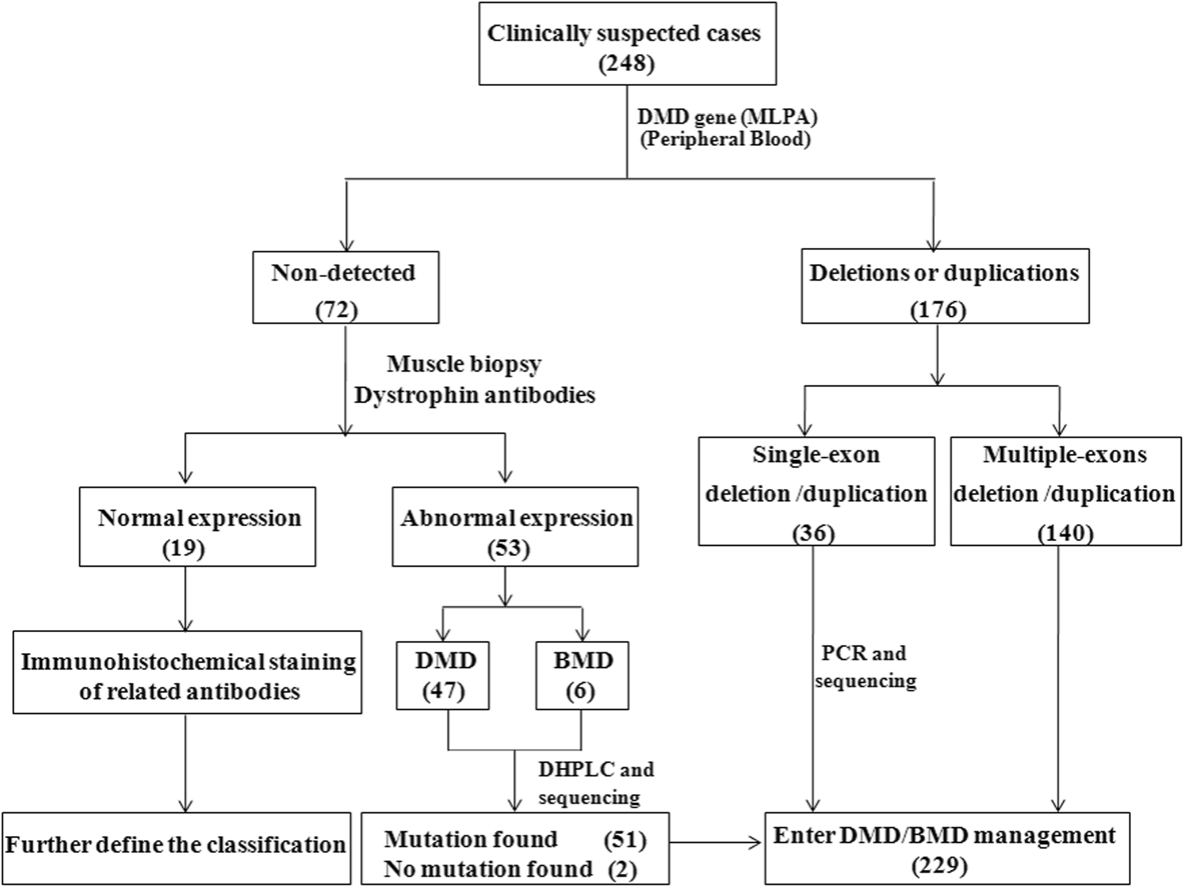
**Diagnostic process for muscular dystrophy in CHFU.** The number in bracket is the total patients in various categories.

Classification of DMD or BMD is determined by neurologists and genetic curators together and based on genetic analysis (reading frame rule) with consideration of clinical information and histopathology including results of dystrophin immunostaining if available (Figure 1).

### MLPA analysis

MLPA (SALSA MLPA probemix P034-A3/P035-A3 DMD/Becker; MRC Holland, Amsterdam, Netherlands) was the first-line method for the identification of mutations in the dystrophin gene, allowing detection of deletions or duplications in all 79 exons [17]. The data was analyzed by Coffalyser MLPA analysis software. For single exon deletion, further verification was carried out by PCR and sequencing.

### DHPLC and sequencing

DHPLC technique was applied to patients with negative MLPA, but with confirmed diagnosis of DMD/BMD by muscle biopsy. After amplification of the 79 primer pairs, unpurified PCR amplicons from patients were mixed in a 1:1 ratio with an aliquot of unpurified PCR amplicon from an unaffected male. The mixture was then denatured, followed by slowly cooling down in a thermal cycler. The results were analyzed by the WAVE nucleic fragment analysis system. The pre-treated amplicons were processed at the optimal separation gradient and temperature determined by WAVEMARKER 4.1 software. If a PCR amplicon presents a chromatogram different from the control in shape or retention time, the corresponding exon was then subjected to direct sequencing to identify the position and the type of mutations using Beckman-coulter CEQ 8000 genetic analyzer. The National Center for Biotechnology Information (NCBI) Gene database (dbSNP) was queried to identify the existence of the common SNPs, and polymerase chain reaction-restriction fragment length polymorphism(PCR-RFLP) was employed to identify the unreported SNPs.

### Dystrophin protein analysis

Muscle biopsies were analyzed by immunofluorescence for dystrophin expression with each of the three NCL-DYS1, NCL-DYS2 and NCL-DYS3 antibodies (Leica Biosystems Newcastle). Patients with dystrophin recognized by NCL-DYS2 against C-terminal region of the protein were considered as BMD [18].

## Results

**Figure 2:**
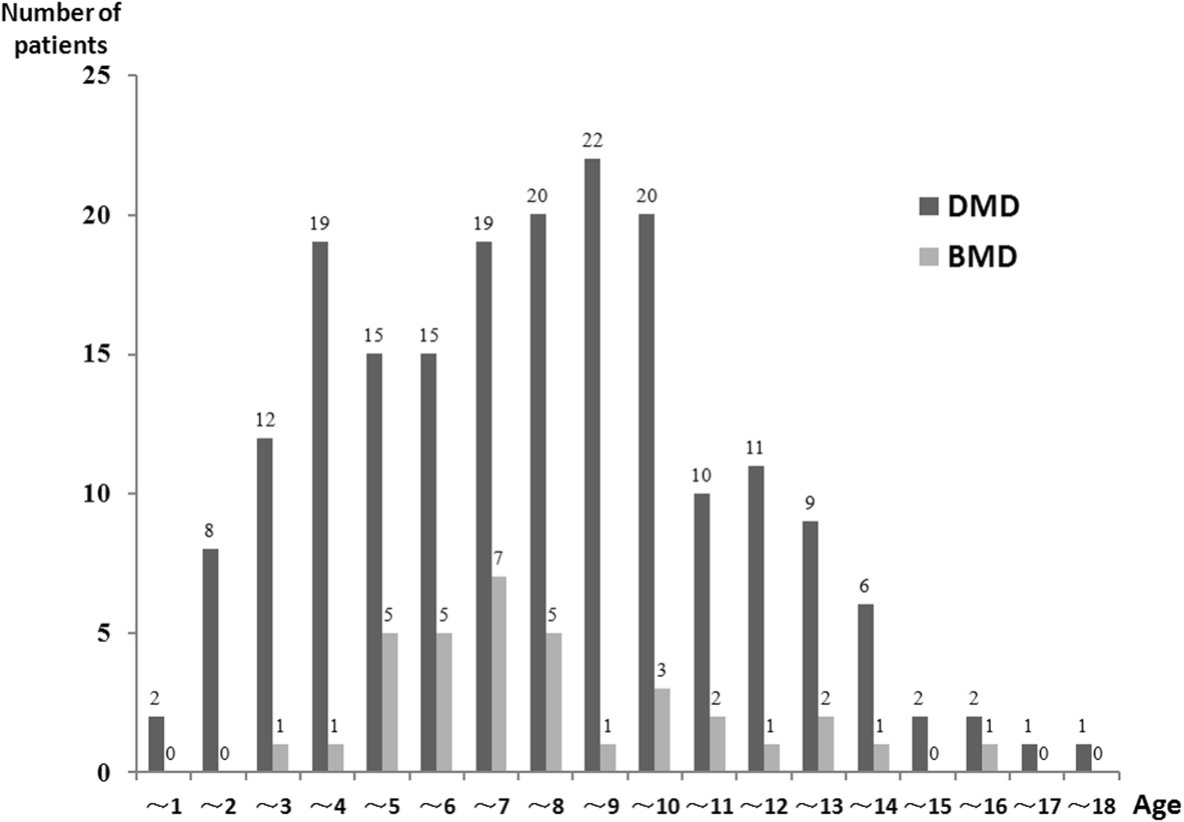
**Ages of registered patients.** All the registrants were under 18 years of age.

**Figure 3:**
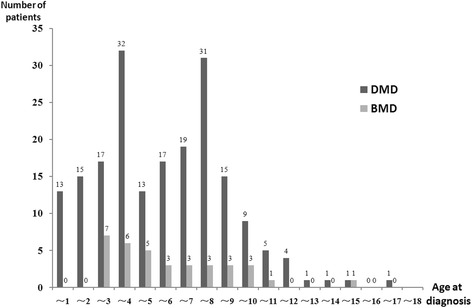
**Age at diagnosis.** 68.1% patients were diagnosed between the age of 2 to 8 years old.

As of December 2013, 229 patients had registered in our database with 194 DMD and 35 BMD with all individuals less than 18 years old (Figure 2). All boys had genetic diagnosis except 2 cases and 71.2% of patients had a muscle biopsy. Diagnosis was made with higher proportion during their age of 3–4 (16.6%) and 7–8 (14.8%) years (Figure 3).

**Figure 4:**
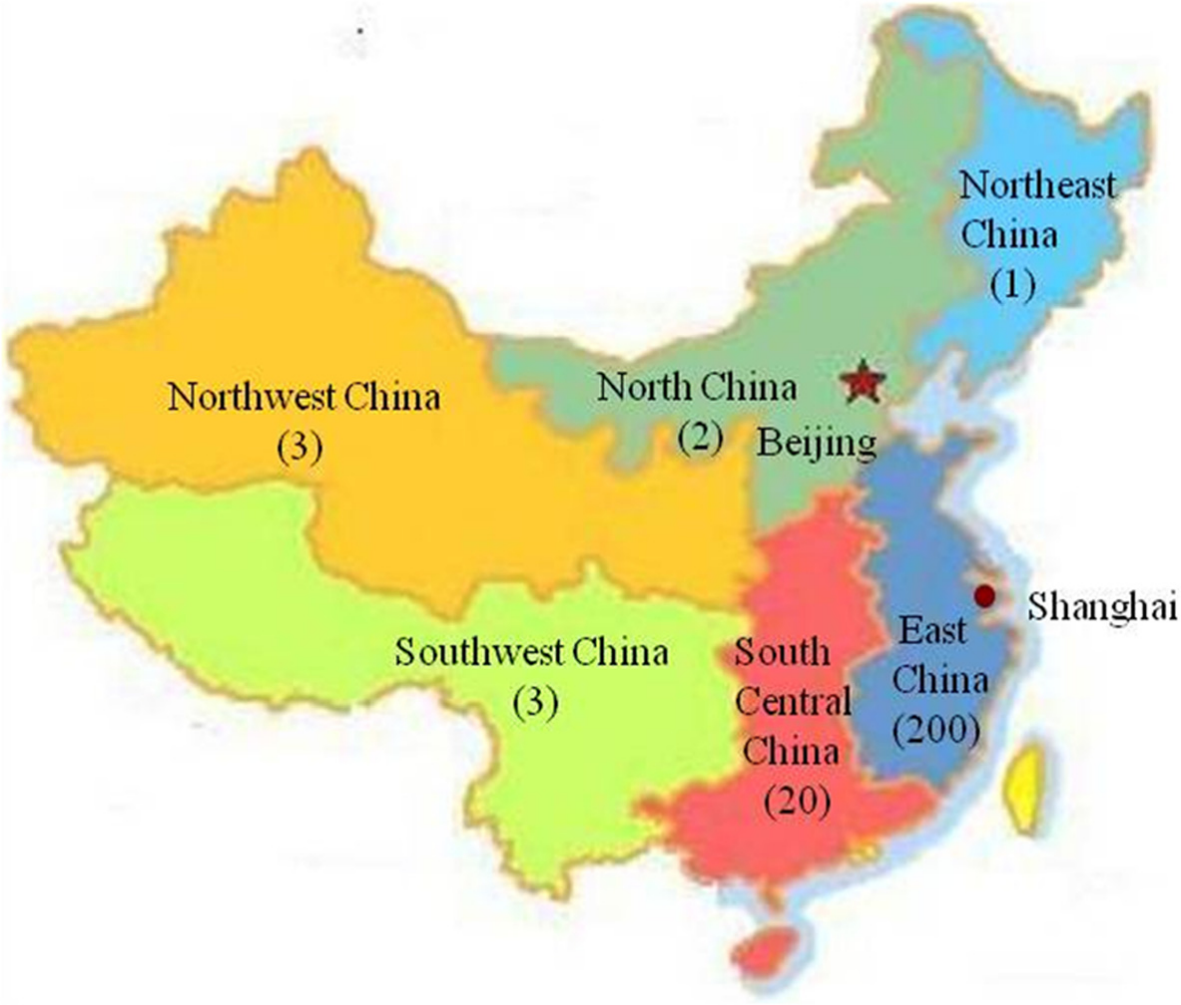
**Geographical distribution of 229 registrants.** The number in bracket is the total patients from the region.

Most patients were from East China including Shanghai (26) and the provinces surrounding Shanghai, namely Jiangsu (57), Anhui (45), Zhejiang (30), Jiangxi (27), Fujian (9) and Shandong (6). A small proportion of registrants resided in South Central China (20), Southwest China (3), Northwest China (3), North China (2) and Northeast China (1) (Figure 4).

**Figure 5:**
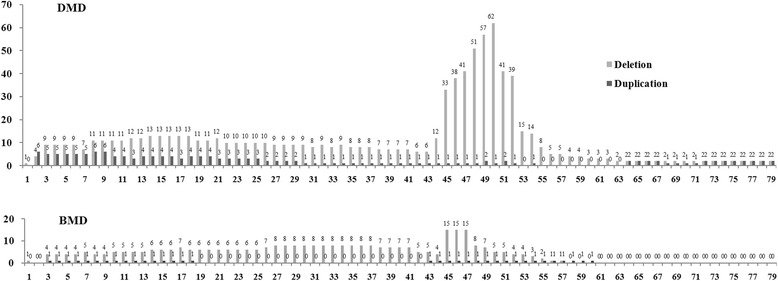
**Frequency of each exon (deletion and duplication) involved in DMD and BMD patients.** Exons 45–52 were the most frequently deleted for DMD patients and deletion of exons 45–47 was the most commonly involved for BMD patients.

As summarized in Figure 5, Table 1 and Table 2, exon deletion was the most frequent (65.5% and 74.3%) followed by point mutations (14.4% and 11.4%), duplications (9.8% and 8.6%) and small insertion/deletion (9.3% and 2.9%) for DMD and BMD, respectively. Two cases with no mutation identified by MLPA and DHPLC were diagnosed as DMD and BMD on the basis of muscle biopsy showing absence of dystrophin expression and clinical manifestation. 33.2% of DMD patients were eligible for exon skipping, among whom 21 (9.2%) and 10 (4.3%) patients being eligible to skipping of exon 51 and 53, respectively (Table 3).

**Table 1:** **Distribution of mutations in DMD/BMD patients**

	**DMD patients**		**BMD patients**	
**Distribution of mutation**	**No. of case**	**% of case**	**No. of case**	**% of case**
Deletion	127	65.5%	26	74.3%
Duplication	19	9.8%	3	8.6%
Deletion and duplication	1	0.5%	0	0
Others*	46	23.7%	5	14.3%
No mutation found**	1	0.5%	1	2.8%
	194	100%	35	100%

*Point mutation, small insertion/deletion mutations and splice site mutations are listed in Table 2. **Diagnosis was made on the basis of absence of dystrophin expression and clinical manifestation.

**Table 2:** **Confirmed point mutations, small deletion/insertion of DMD/BMD patients**

**CHFU no.**	**Phenotype**	**Family history**	**Exon/Intron**	**Sequencing results**	**Change of protein**	**Carrier status of mother**
7	DMD	No	Exo 19	c.2308A > T	p.Lys770X	Unknown
11	DMD	No	Exo 34	*c.4746_4747delCT	p.Leu1583ThrfsX18	Unknown
12	DMD	No	Exo 19	c.2302C > T	p.Arg768X	Unknown
13	DMD	Yes	Exo 34	*c.4808_4809insGGAA	p.Met1603GlufsX3	Yes
19	DMD	Yes	Exo 22	c.2816 T > A	p.Leu939X	Yes
20	DMD	No	Exo 17	c.2137C > T	p.Gln713X	Unknown
21	DMD	No	Exo 51	*c.7327_7328insA	p.Asn2444SerfsX9	Unknown
22	DMD	Yes	Int 21	c.2803 + 1G > A		Yes
26	DMD	No	Exo 33	c.4630delA	p.Glu1545fs	Unknown
33	DMD	No	Exo 16	*c.1886C > G	p.Ser629X	Unknown
42	DMD	Yes	Exo 60	c.8944C > T	p.Arg2982X	Yes
45	BMD	No	Int 17	*c.2169-1G > T		Unknown
59	DMD	No	Exo 52	c.7657C > T	p.Arg2553X	Unknown
60	DMD	No	Exo 55	*c.8147-8148insCAGAAG	p.His2717ArgfsX3	Unknown
				CTGAAACAACTGCCAA		
				TGTCCTACA		
61	DMD	No	Exo 13	c.1594C > T	p.Gln532X	Yes
62	BMD	Yes	Exo 4	*c.264_264 + 4delTGTAA	p.Pro63_Asn88del	Yes
64	DMD	Yes	Exo 64	*c.9297_9300dup	p.Asn3100Asnfs	Yes
67	BMD	No	Exo 25	c.3432G > T	p.Gln1144His	Unknown [19]
68	DMD	No	Exo 18	*c.2191delC	p.Arg1288LeufsX7	Unknown
71	DMD	No	Exo 44	c.6292C > T	p.Arg2098X	Yes
74	DMD	No	Int 67	c.9807 + 5G > A		Unknown
75	DMD	No	Exo 42	*c.6045delA	p.Asp2016AsnfsX23	Unknown
79	DMD	No	Exo 64	c.9337C > T	p.Arg3113X	Unknown
80	BMD	No	Exo 25	*c.3388G > T	p.Glu1130X	Unknown
86	DMD	No	Exo 66	c.9568C > T	p.Arg3190X	Unknown
89	DMD	No	Exo 33	*c.4538-4541GTGAdel	p.Lys1514fs	Unknown
91	DMD	No	Int 54	*c.8028-1G > C		Unknown
92	DMD	Yes	Exo 67	*c.9722_9723delCT	p.Tyr3242ProfsX8	Yes
93	BMD	No	Exo 29	c.3940C > T	p.Arg1314X	Unknown
96	DMD	Yes	Exo 47	*c.6804_6807delACAA	p.Asn2269fs	Yes
97	DMD	Yes	Exo 59	c.8713C > T	p.Arg2905X	Yes
100	DMD	No	Exo 44	*c.6391_6392delCA	p.Asn2132Leufs	Unknown
101	DMD	Yes	Exo 10	c.998C > A	p.Ser333X	Yes
106	DMD	No	Exo 55	c.8038C > T	p.Arg2680X	Unknown
107	DMD	No	Exo 15	*c.1713insT	p.Phe572fs	No
113	DMD	No	Exo 64	c.9337C > T	p.Arg3113X	Unknown
114	DMD	No	Exo 42	*c.6033insTTAA	p.Leu2012IlefsX11	Yes
127	DMD	No	Exo 19	*c.2300delA	p.Gly768GlufsX25	Unknown
135	DMD	Yes	Exo 59	*c.8740G > T	p.Glu2914X	Yes
145	DMD	Yes	Exo 70	c.10171C > T	p.Arg3391X	Yes
148	DMD	No	Exo 52	c.7657C > T	p.Arg2553X	Unknown
161	DMD	No	Exo 10	*c.1033C > T	p.Gln345X	Yes
165	DMD	No	Exo 32	c.4483C > T	p.Gln1495X	Unknown
170	DMD	Yes	Exo 20	*c.2571delC	p.Pro858ProfsX13	Yes
171	DMD	No	Int 14	c.1704 + 1G > C		Yes
172	DMD	No	Exo 44	c.6292C > T	p.Arg2098X	Yes
177	DMD	Yes	Exo 12	*c.1412delC	p.Lys472GlyfsX15	Yes
192	DMD	No	Exo 70	c.10108C > T	p.Arg3370X	Unknown
193	DMD	No	Exo 39	*c.5485C > T	p.Gln1829X	Unknown
197	DMD	Yes	Exo 64	*c.9297_9300dup	p.Asn3100Asnfs	Yes
201	DMD	No	Exo 70	c.10108C > T	p.Arg3370X	Unknown

*Abbreviations:* *BMD* Becker muscular dystrophy, *DMD* Duchenne muscular dystrophy, *CHFU* Children’s Hospital of Fudan University.

Asterisks indicate novel mutations that have not been previously reported in the Leiden Muscular Dystrophy database (updated 30 December 2013) (http://www.dmd.nl/).

Case no.64 is no.197’s uncle.

**Table 3:** **Candidates for 51 and 53 exon skipping for restoration of dystrophin expression (source:** **www.clinicaltrials.gov** **)**

**51 exon skipping**			**53 exon skipping**		
**Deleted exon(s)**	**No. of individuals**	**% of registrants**	**Deleted exon(s)**	**No. of individuals**	**% of registrants**
45-50	8	3.5%	52	3	1.3%
47-50	2	0.9%	47-52	1	0.4%
48-50	4	1.7%	48-52	3	1.3%
49-50	2	0.9%	49-52	2	0.9%
50	2	0.9%	50-52	1	0.4%
52	3	1.3%			
52-63	0	0%			
Total	21	9.2%		10	4.3%

Table 4 described the clinical characteristics of DMD/BMD patients included in the registry. Among the DMD registrants, 82.5% of boys were ambulant whereas all the BMD registrants were ambulant. DMD patients lost their walking ability from 9 to 12 years old. 25.3% of DMD boys were receiving steroid treatment. Cardiac functions were examined for 46.4% DMD boys and 45.7% BMD boys and respiratory functions were examined only for 18.6% DMD boys and 14.3% BMD boys. The low percentage was related to the young age of the majority of registrants. No abnormalities in cardiac functions were detected in 44.3% of DMD and 45.7% of BMD patients tested. Among the boys with cardiac dysfunction (diagnosed as dilated cardiomyopathy), three boys with significant deficiency in left ventricular ejection fraction (25% to 48 % normal levels) and one boy with abnormal ECG (electrocardiogram) were prescribed with ACE–inhibitor treatment (1) and the ACE–inhibitor and β-blocker (3). None of the patients used ventilator support or received scoliosis surgery.

**Table 4:** **Walking capability, medications and intervention in the registrants with DMD and BMD**

	**DMD patients**		**BMD patients**	
	**No. of case**	**% of case**	**No. of case**	**% of case**
**Walking capability**				
Normal walking	160	82.5%	35	100%
Not able to walk	34	17.5%	0	0
	194	100%	35	100%
**Cardiac function**				
Normal	86	44.3%	16	45.7%
Dysfunction	4	2.1%	0	0
Not performed	104	53.6%	19	54.3%
	194	100%	35	100%
**Respiratory function**				
Normal	36	18.6%	5	14.3%
Dysfunction	0	0	0	0
Not performed	158	81.4%	30	85.7%
	194	100%	35	100%
**Steriod use**				
Current	49	25.3%	2	5.7%
Used to	2	1%	0	0
Never	143	73.7%	33	94.3%
	194	100%	35	100%

## Discussion

Establishing a comprehensive database for DMD and BMD requires wide collaborations and financial support. With the strong support from Children’s Hospital of Fudan University, we have been able to build a preliminary multidisciplinary professional team including paediatric neurologist, rehabilitation specialist, physical therapist, paediatric heart specialist, paediatric pulmonary specialist, paediatric orthopaedist, geneticist and psychologist [6]. Currently, diagnosis has been made chiefly in our Molecular and Pathological Diagnostic Laboratory responsible for genetic testing and immunohistochemical analyses of muscle biopsy. This is coordinated by our DMD Outpatient Clinic, which opened in January 2013 and focuses on registry of clinical data, treatment regime and follow-up. Since then, an increasing number of DMD/BMD patients were diagnosed and registered in the hospital. We expect that our database will soon contain the largest number of DMD/BMD patients in China and one of the largest in the world. This will enhance the accuracy of diagnosis, help in developing effective treatment and facilitate future clinical trials.

### Age at diagnosis

Most of the registrants within the CHFU database were diagnosed during the age of 3–4 (16.6%) and 7–8 (14.8%) years. The high rate of diagnosis at 3–4 year-old group is the result of health screening for children entering Nurseries and Kindergartens. However, the 7–8 year-old children visited the outpatient clinic for clinical symptoms typical for muscular dystrophy such as toe walking and difficulty to climb stairs. Most boys of this age group were from rural areas, indicating the lack of awareness of the disease by both parents and primary care doctors. The data raises the urgent need for health authority and medical institutions to take initiative to educate the public, especially in rural areas to improve early diagnosis and provide preventive care and optimal management.

### Mutation distribution and genotype/phenotype correlation

The large number of DMD/BMD patients in the CHFU database also revealed the specific pattern of mutations in the Chinese population. In this study, we analyzed the distribution of individual exons based on their frequency of deletion or duplication. Our data indicate that exon deletion is the most frequent followed by point mutations, duplications and small insertion/deletion. In DMD patients, exons 45–52 are the most frequently deleted. This is similar to the pattern described in other databases [16,17,20] and those of previously reported in Chinese population [21-23]. Similarly, the most common deletion in BMD patients was exons 45–47 (22.9%) [17]. In general, the severity of the phenotype correlates to the reading-frame rule [24]. Similar to the report by Sylvie Tuffery-Giraud et al. [20], our database shows 95.2% of DMD patients had out of frame or nonsense mutations whereas 85.7% of the BMD mutations were in-frame, consistent with two previous studies in Chinese patients with DMD/BMD [21,22]. The phenotype for in-frame mutations is to some extent determined by the location and the size of the deletion. Large in-frame deletions removing up to 35 exons in the central rod domain have been described for patients with relatively mild BMD [25,26]. Within our database, the muscle biopsy of a patient with a large in-frame deletion of exons 27–57 showed absence of dystrophin expression using antibody NCL-DYS1 (against amino acids 1181–1388), whereas near normal level signals were detected using antibody NCL-DYS2 and NCL-DYS3 (against amino acids 3669–3685 and 321–494 respectively). Another patient with a large in-frame deletion of exons 10–41 showed absence of dystrophin expression using antibody NCL-DYS1 and NCL-DYS3, whereas near normal level signals were detected using antibody NCL-DYS2. Both patients aged 8 years. No detectable muscle weakness was found and the only pathological feature was the elevation of serum creatine kinase (CK) (1926 U/L and 12350 U/L respectively). Most point mutations are nonsense mutations resulting in DMD. However, there were 2 BMD cases with point mutations in our database (Seen in Table 2). One 5-year-old patient with the mutation c.3940C > T had high serum CK levels (11740 U/L) as the only clinical abnormality. Muscle biopsy showed lack of dystrophin using antibody NCL-DYS1 against an epitope encoded by exon 29, but positive staining using antibody NCL-DYS2 and NCL-DYS3, consistent with the previous report of a BMD family [27]. The other 4-year-old boy with the novel point mutation (c.3388G > T) showed the clinical abnormality of bringing the second foot up to join the first rather than going foot over foot when climbing stairs and high serum CK levels (9100 U/L). The muscle biopsy showed lack of dystrophin using antibody NCL-DYS1, NCL-DYS2 and NCL-DYS3. We also found a large BMD pedigree with 4 patients and 10 carriers of a novel frame-shift mutation in exon 4 in this family. The 5-year-old proband showed only mild atrophy of bilateral quadriceps, without symptomatic weakness. Muscle biopsy revealed mild myopathic features including variation in fiber size and a few necrotic and regenerating muscle fibers. Immunohistochemical analysis demonstrated decreased expression with antibody NCL-DYS3 and near normal expression with antibody NCL-DYS1 and NCL-DYS2. Serum CK levels were consistently around 2854 U/L. The oldest patient aged 35 was diagnosed at the age of 9 and lost walking ability in his thirties. The other two patients aged 27 and 23 had the onset of the disorder around 10 years old with difficulty in walking and climbing stairs. All the patients revealed bilateral atrophy limited to quadriceps femoris without hypertrophy of the calves. We considered it as quadriceps myopathy, a clinical variant form of BMD [28-30].

### Family history and genetic counseling

In our study, 23.1% of the probands had a family medical history of the disease and several pedigrees also had a large number of patients and mutation carriers, especially in less developed areas. Through patient registry, we provided information on genetic counseling and prenatal diagnosis for pedigrees and those who had given birth to DMD/BMD babies to prevent this disease. 15 healthy children have been born by prenatal diagnosis in these families in 2013. Genetic counseling is therefore valuable for prenatal diagnosis and vitally important to those big families in less developed areas where literacy level is relatively low and lack of knowledge about genetic diseases.

### Steroid treatment and cardiac and pulmonary management

26.3% of DMD patients in the database were treated with steroids. This rate was relatively low compared to that in the developed countries [16,31,32]. The main reason for this is due to the lack of awareness of physicians specialized in treating muscular dystrophies. The Family Guide for the Diagnosis and Management of Duchenne Muscular Dystrophy [33] became available in our hospital only in January 2013, when DMD Outpatient Clinic was opened. However, this environment is changing with the establishment of the database and the adoption of the TREAT-NMD project guidelines [6,34]. A steady increase in the use of steroid treatment is expected in China. As to the evaluation of cardiac and pulmonary function, we refer to the Family Guide, which indicates that baseline evaluation of cardiac function needs to be performed at the confirmation of the diagnosis or at latest by the age of six years. Evaluation includes an electrocardiogram (ECG) and echocardiogram and occurs at least once every two years until the age of ten. Yearly complete cardiac evaluations begin at approximately ten years of age or at the onset of cardiac signs and symptoms, if earlier. If noninvasive cardiac tests show abnormalities, increased surveillance is required, at least every six months, and drug treatment will be initiated by our cardiologists. In our database, four boys were prescribed for treatment with ACE–inhibitor (1) and the ACE–inhibitor and β-blocker (3). One of them has an abnormal ECG that showed right ventricular hypertrophy and sinus tachycardia and the others have abnormal echocardiograms (ejection fraction ranging from 25-48% of normal levels) [31]. Minimal assessment of pulmonary function (such as measurement of forced vital capacity [FVC] at least annually) allows the child to become familiar with the equipment and the team to assess the maximum respiratory function achieved, while a boy with DMD is still walking. The main emphasis of pulmonary assessment is after the loss of independent walking [33]. However, pulmonary management is far from perfect in China. In our database, none of the patients used ventilator support. No abnormality has been found in the patients who received pulmonary function tests, whereas most wheelchair-bound patients are taken care of by their family members at home and are reluctant to get tested. This is largely due to two factors: lack of awareness by physicians of significance of these measures to the quality of patient’s life and lack of medical insurance to cover the expenses in China. This reinforces the urgency to raise awareness of the disease to the public, physicians and governmental organizations.

## Conclusions

The CHFU database is the most comprehensive one in China and has the capacity to understand the natural history of DMD/BMD and facilitate international cooperation in developing experimental therapies. This is especially important for clinical trials such as exon skipping targeting only a subset of the patient population. For example, international cooperation is essential for any future clinical trials of exon skipping targeting exons only applicable to less than 5% of DMD population. A detailed database of patients is critical for providing a quick selection of patients based on a pre-determined genotypic and phenotypic criteria. In about two years, 229 DMD/BMD male patients have registered in our database and 9.2% and 4.3% patients are eligible for clinical trial focused on ‘skipping’ exon 51 and 53, respectively. 11.4% DMD/BMD male patients were found to be suitable for the read-through of nonsense mutations trial. Given the size of the population in China and the rate of incidence, we hope, by extending the established CHFU database, we will be able to make significant contribution to the development of clinical trials and to promote therapeutic research in China.
